# Supplementary material for: Periductal bile acid exposure causes cholangiocyte injury and fibrosis
Source: PLoS One. 2022 Mar 16;17(3):e0265418. doi: 10.1371/journal.pone.0265418 (PMC8926245; doi:10.1371/journal.pone.0265418)
Supplement: Attachment — Submitted filename: Response to Reviewers.docx [file pone.0265418.s001.docx]

## Emily Chenette

## Editor-in-Chief

## *PLOS ONE*

February 2022

Dear Dr. Chenette,

Enclosed, please find our revised manuscript entitled **"Periductal bile acid exposure causes cholangiocyte injury and fibrosis,"** which we are resubmitting to PLOS ONE.

We are grateful to the editor for the opportunity to revise our article. As advised, we have revised the manuscript based on the helpful comments and suggestions provided by the reviewers. Please find below a detailed point-by-point response to each of the reviewers.

- We ensured that our manuscript meets PLOS ONE's style requirements.
- We removed any funding-related text from the manuscript.
- The dataset can be found at:

URL: <https://www.kaggle.com/miridotan/periductal-bile-acid-exposure>

DOI: 10.34740/kaggle/dsv/3208167

- The manuscrip was uploaded as a pre-print to: <https://www.biorxiv.org/content/10.1101/2022.02.16.480650v1>
- The images were uploaded and adjusted using Preflight Analysis and Conversion Engine (PACE) digital diagnostic.
- Below you will find a point by point response to each of the points made by the reviewer.

Thank you in advance for considering our revised manuscript. We hope that it will now prove acceptable for publication in PlosOne.

Sincerely,

Orith Waisbourd-Zinman

oritwz@gmail.com

+972-54-2077679

Division of Gastroenterology, Nutrition and Liver Diseases

Schneider Children's Medical Center of Israel

Response to reviewers:

Reviewer 1:

1. First and foremost, two preliminary remarks should be made here: biliary atresia (BA) is an inflammatory process of the intra- and extrahepatic biliary tree, leading to ongoing fibrosis and deteriorating liver function. However, according to diagnostic patterns and surgical treatment, BA is only defined by the pathological findings along the extrahepatic bile ducts. This contradiction is frequently overseen, when etiologically directed research is performed.

**Response:** We thank the reviewer for this comment and agree that BA is a pan-cholangiopathy. At the time of BA diagnosis the extra-hepatic biliary tree is more effected compared to the intra-hepatic bile ducts; we thus hypothesize that the extra-hepatic cholangiocytes are either more sensitive to injury in the neonate or that there are hepatic mechanism compensating in part for the intra-hepatic cholangiocytes. Here in this study, we specifically aimed to determine the effect of increased permeability of the extra-hepatic bile ducts both to cholangiocytes and to the peri-ductal area. Based on the reviewer suggestion to modify the paper to reflect cholangiopathies in general and not specifically BA, we have edited the text accordingly.

2. The second point is that there is so far no BA-animal model available, which displays precisely the pathomechanism and the course of the human disease. Taking this consideration into account, one should act cautiously when findings in mouse models or cell cultures shall be translationally interpreted.

**Response:** We thank the reviewer for this comment. We agree with this limitation of the study. Indeed mice research should be interpreted cautiously with future translational work to support those findings both from our labs and other groups. We have emphasized this well taken point in the discussion.

3. The authors of the submitted study show that bile acid exposure to isolated extra hepatic bile ducts, which were harvested from adult and neonatal Balb/c-mice, results in cholangiocyte injury and fibrosis. They also demonstrate that ursodeoxycholic acid has a protective effect and reduces the destruction of the epithelial layer of the bile ducts. Limitations of the study are listed by the authors themselves, of which the argument concerning the exposure of the specimens to the bile salts was not restricted to the epithelium but to the bile duct in toto, is the most relevant. The relevance of the observations could be improved by showing that no isolated intra-hepatic bile ducts react the same way.

**Response:** This is indeed an important point. We are not aware of a methodology that isolates intra-hepatic bile ducts as a whole rather than single cholangiocytes, and are not caplbe of performing it in our facility. Our study specifically aimed to determine the effect of bile acids exposure to the extra hepatic peri-ductal area and cholangiocytes in the basolateral side. We agree that the results do no imply on intra-hepatic bile ducts and we have refied our discussion to include that.

4. Although the study provides interesting results about the toxic effect of bile salts to bile ducts and the already demonstrated protective value of ursodeoxycholic acid, it is not acceptable to discuss these findings in the context of the pathomechanism of BA. Hence, the observations of this interesting study should be imbedded in any other context. **Response:** We thank the reviewer for this comment. We have accepted this comment in full and substantially reduced the context of biliary atresia throughout the manuscript and adjugsted the discussed the findings in the context of cholangiopathies in general.
